# Cortical imbalance following delayed restoration of bilateral hearing in deaf adolescents

**DOI:** 10.1002/hbm.25875

**Published:** 2022-04-15

**Authors:** Carly A. Anderson, Sharon L. Cushing, Blake C. Papsin, Karen A. Gordon

**Affiliations:** ^1^ Archie's Cochlear Implant Laboratory The Hospital for Sick Children Toronto Ontario Canada; ^2^ Neurosciences and Mental Health, SickKids Research Institute Toronto Ontario Canada; ^3^ Department of Otolaryngology—Head and Neck Surgery The Hospital for Sick Children Toronto Ontario Canada; ^4^ Department of Otolaryngology—Head and Neck Surgery University of Toronto Toronto Ontario Canada; ^5^ Present address: Institute of Cognitive Neuroscience University College London London UK

**Keywords:** auditory development, bilateral processing, binaural, cortical plasticity, deafness, electroencephalography, hemispheric asymmetry, longitudinal, pediatric, sequential cochlear implantation

## Abstract

Unilateral auditory deprivation in early childhood can lead to cortical strengthening of inputs from the stimulated side, yet the impact of this on bilateral processing when inputs are later restored beyond an early sensitive period is unknown. To address this, we conducted a longitudinal study with 13 bilaterally profoundly deaf adolescents who received unilateral access to sound via a cochlear implant (CI) in their right ear in early childhood before receiving bilateral access to sound a decade later via a second CI in their left ear. Auditory‐evoked cortical responses to unilateral and bilateral stimulation were measured repeatedly using electroencephalogram from 1 week to 14 months after activation of their second CI. Early cortical responses from the newly implanted ear and bilateral stimulation were atypically lateralized to the left ipsilateral auditory cortex. Duration of unilateral deafness predicted an unexpectedly stronger representation of inputs from the newly implanted, compared to the first implanted ear, in left auditory cortex. Significant initial reductions in responses were observed, yet a left‐hemisphere bias and unequal weighting of inputs favoring the long‐term deaf ear did not converge to a balanced state observed in the binaurally developed system. Bilateral response enhancement was significantly reduced in left auditory cortex suggesting deficits in ipsilateral response inhibition of new, dominant, inputs during bilateral processing. These findings paradoxically demonstrate the adaptive capacity of the adolescent auditory system beyond an early sensitive period for bilateral input, as well as restrictions on its potential to fully reverse cortical imbalances driven by long‐term unilateral deafness.

## Graphical abstract

Bilateral auditory processing deficits are pervasive and restrictive in deaf cochlear implant patients, but the cortical mechanisms remain poorly understood. This longitudinal electroencephalogram study identified a persistent cortical imbalance towards the long‐deprived ear driven by delayed restoration that disrupts bilateral integration mechanisms.

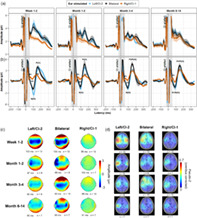

**Abbreviations**
AC: auditory cortex
CI: cochlear implant
CI‐1: first cochlear implant
CI‐2: second cochlear implant
CI‐B: bilateral cochlear implant
EMMs: estimated marginal means
GFP: global field power
LMM: linear mixed model
PZ: pseudo‐Z
TRACS: Time‐Restricted Artifact and Coherent source Suppression

## 1 INTRODUCTION

Unilateral auditory deprivation early in life can lead to a strengthening of connections from the stimulated side and an overrepresentation of the hearing ear in the central auditory system (Gordon et al., 2013, 2015; Kral, 2013; Moore, 1994; Popescu & Polley, 2010; Tillein et al., 2016). Although the brain has a remarkable ability to adapt to sensory loss (Knudsen, 1988; Lickliter, 2000), functional reorganizations following deafness in early life (Kral et al., 2016; Lomber et al., 2010; Meredith et al., 2011) can be long term and somewhat irreversible (Easwar et al., 2017; Gordon et al., 2013; Lee et al., 2020). Deafness‐related plasticity can differentially impact the success of future auditory restoration with a neuroprosthetic cochlear implant (CI) as shown in adults (Anderson et al., 2017, 2019; Lazard et al., 2013; Sandmann et al., 2012). Because access to auditory inputs from both ears forms the basis of spatial hearing, which is crucial for locating and distinguishing separate sound sources and understanding speech in noisy environments, spatial hearing is compromised in children with unilateral hearing (Litovsky & Gordon, 2016; Reeder et al., 2015). They are also at risk of poorer speech, language, and educational outcomes compared to their hearing peers (Fischer & Lieu, 2014; Lieu et al., 2010). For children with profound bilateral hearing loss, clinical guidelines thus recommend providing access to sound via CIs in both ears simultaneously to avoid unilateral hearing and promote typical cortical function and improved hearing outcomes (Gordon et al., 2013, 2015; Health Quality Ontario, 2018; Ramsden et al., 2012). However, children with bilateral deafness were traditionally provided with only one CI and so developed with unilateral auditory stimulation in one ear, and unilateral deprivation in the other. Following evidence of the significant cortical (Gordon et al., 2013) and functional benefits (Chadha et al., 2011; Litovsky & Gordon, 2016; Van Deun et al., 2010) of providing access to sound in both ears, these children were provided with a second sequential CI in the deaf ear following an inter‐implant delay.

The impact of delayed bilateral restoration to an auditory system that has developed under unilateral conditions has been shown in the unilateral pathways (Gordon et al., 2013; Jiwani et al., 2016; Lee et al., 2020; Polonenko et al., 2017, 2018a). However, the impact on the bilateral pathways and whether these unilaterally driven cortical consequences can be wholly or even partially reversed, particularly during adolescence, is unknown. Here we examined: (i) the impact of long‐term unilateral deprivation/stimulation in early development on bilateral auditory processing following later auditory restoration in adolescence, and (ii) longitudinal trajectories of change in auditory processing over the first year of bilateral restoration in adolescence. Electrophysiological evidence in both children and animal models indicates that the earliest latency peaks in the auditory‐evoked potential, including P1 (Gilley et al., 2008; Sharma et al., 2002, 2005) and N1 (Ponton & Eggermont, 2001), are vulnerable to the effects of auditory deprivation and can be a marker for auditory pathway maturation and plasticity following cochlear implantation (Ponton & Eggermont, 2001). We therefore interrogated the earliest identifiable component to examine the fundamental integrity of the auditory pathways at an early stage of cortical processing that presents the first bottleneck to successfully achieving subsequent higher level auditory processing, such as speech perception, when listening with CIs.

When the brain integrates sensory inputs, it maintains balance and avoids over representation of one input via excitation–inhibition mechanisms (Mariño et al., 2005; Wehr & Zador, 2003). Animal models indicate that balanced inhibition is a key mechanism in effective bilateral integration of sound inputs from each ear (Brand et al., 2002; Grothe, 2003; Pouille, 2001; Wehr & Zador, 2003) but that this is disrupted by auditory deprivation (Vale et al., 2004; Vale & Sanes, 2002) with adverse effects including increased excitability and decreased inhibition in primary auditory cortex neurons (Kotak et al., 2005; Kral et al., 2005; Tillein et al., 2016). These alterations to neuronal networks may manifest as the bilateral processing difficulties we continue to see in children with CIs, including reduced binaural inhibition in the auditory brainstem (Gordon et al., 2008, Gordon, Chaikof, et al., 2012; Gordon, Salloum, et al., 2012; Steel et al., 2015), reduced functional connectivity between the left and right auditory cortex (Smieja et al., 2020), and decreased ability to perceive sounds from both ears as one fused percept (Steel et al., 2015). When listening with both ears, normal hearing adults show a greater suppression of ipsilateral compared to contralateral responses in both hemispheres (Kaneko et al., 2003). Complementary to this, we have observed that bilateral stimulation evokes greater activity compared to unilateral stimulation in the ipsilateral but not contralateral auditory cortex in normal hearing children (Easwar et al., 2017; Yamazaki et al., 2018). In each hemisphere, suppression of inputs from the ipsilateral ear may enable typical contralateral dominance of the auditory system to prevail, supporting equal weighting and cortical representation of inputs for successful bilateral integration (Easwar et al., 2017; Fujiki et al., 2002). This has led us to ask whether unilaterally driven development leads to cortical imbalances in the auditory system that compromise its ability to equally weight and integrate inputs from both ears when bilateral inputs are later restored.

Unilaterally driven consequences may be further compounded in children and adolescents who have experienced a long inter‐implant delay and receive bilateral access to sound after the close of an early sensitive period. Collectively, animal models and data from deaf children have demonstrated the existence of early auditory sensitive periods for cochlear implantation, suggesting that stimulation should be provided in early childhood within a period of approximately 3.5–5 years of auditory deprivation when the central auditory pathways are maximally plastic (Gordon et al., 2005; Kral et al., 2001; Lee et al., 2001; Oh et al., 2003; Sharma et al., 2002). Decreased and delayed synaptic development and deficits in cortico–cortico interactions due to the absence of auditory stimulation are, amongst others, some of the neural mechanisms thought to contribute to the close of auditory sensitive periods by pre‐school ages for cochlear implantation (review in Kral, 2013; Kral & Sharma, 2012). When binaural auditory experience is disrupted by unilateral hearing loss during an early sensitive period, distinct aspects of neuronal sensitivity in the primary auditory cortex that support binaural integration are disrupted, even when the hearing loss is mild and short‐term (Polley et al., 2013). Central representations of the hearing ear are strengthened and for the deprived ear are weakened in animal models (Kral, Heid, et al., 2013; Kral, Hubka, et al., 2013; Popescu & Polley, 2010; Tillein et al., 2016) and in children (Gordon et al., 2013, 2015; Jiwani et al., 2016). A cross‐sectional study suggested that such unilaterally driven consequences can be mitigated by providing a second CI within 1.5 years of the first CI and may not be possible to fully reverse if bilateral input is provided beyond this sensitive period for binaural input (Gordon et al., 2013). However, impact of early unilateral deprivation/stimulation and delayed bilateral restoration on bilateral processing, and whether unilaterally driven reorganizations can be reversed in adolescence, remains unknown. Although adolescence is a later phase of life occurring after the close of early auditory sensitive periods, it is wide‐spanning (10–24 years, Sawyer et al., 2018) and known as an extended period of dynamic and individually variable cortical change (Foulkes & Blakemore, 2018; Giedd, 2004; Giedd et al., 1999; Steen et al., 1997).

Having previously identified contralateral strengthening of unilateral pathways from the first implanted ear (Gordon et al., 2013; Jiwani et al., 2016) and an early sensitive period for provision of bilateral input (Gordon et al., 2013), we hypothesized that: (i) long‐term unilateral deprivation/stimulation would disrupt bilateral processing, with a cortical imbalance between new and established inputs driven by an asymmetric weighting toward the strengthened first implanted ear, and (ii) there would be little change in cortical processing of new auditory input over time and an incomplete reversal of the effects of unilaterally driven development.

## 2 MATERIALS AND METHODS

### 2.1 Participants

**FIGURE 1:**
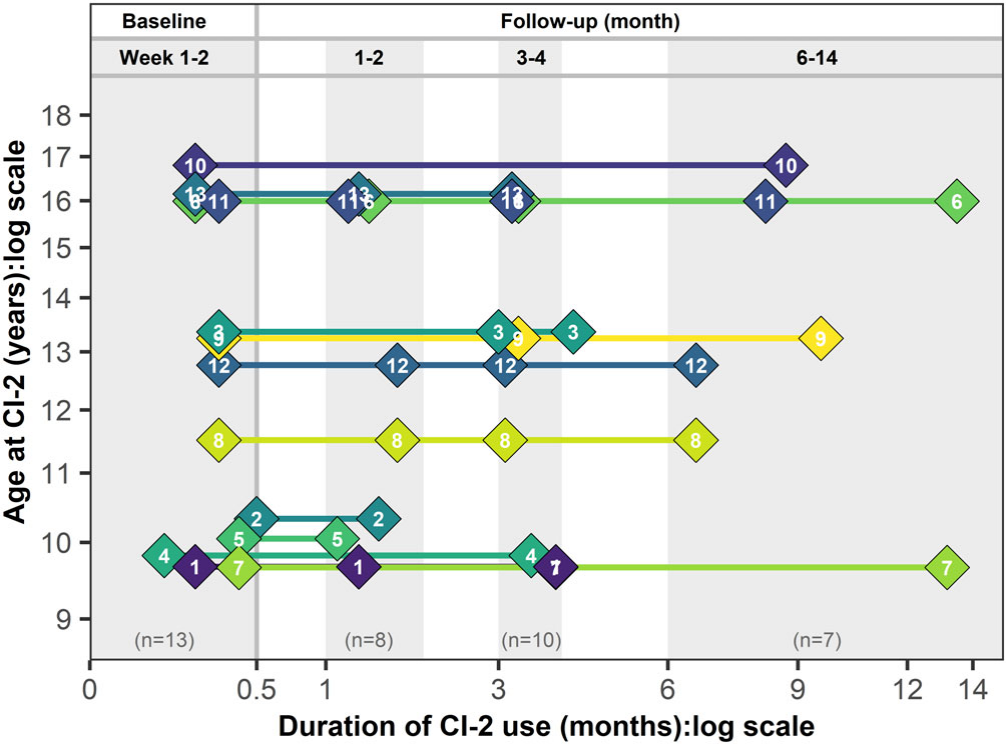
Timeline of EEG recordings for each participant. Participants completed multiple EEG recording sessions over the first year of bilateral CI use. All participants had a “baseline” recording at 1–2 weeks following initial activation of their second CI, and at least one “follow‐up” recording spanning from 1 to 14 months of bilateral CI use. The four categorical time groupings used in subsequent analyses are indicated by shaded boxes and labels. Points are color‐coded and labeled by participant number that are ordered by age at CI‐1. Note that for Participant #3, only their Month 3 data were included in analyses where time was treated as a categorical factor. Both Months 3 and 4 data were included in supplementary modeling when time was treated as a continuous factor

We conducted a prospective clinical cohort study with sample size determined by the available clinical sample of deaf adolescents receiving cochlear implants sequentially at the Hospital for Sick Children. A group of 15 pre‐/adolescents (9–18 years old) were identified from a larger cohort (Jiwani et al., 2016) (see Data S1) based on the following criteria: (i) all had early onset bilateral deafness and received one CI in their right ear before 5 years of age (right/CI‐1); (ii) all were successful unilateral implant users and did not receive auditory input via a hearing aid or CI in their opposite ear; (iii) all later received a second CI in the opposite left ear after 9 years of age (left/CI‐2), with patients experiencing approximately a decade of unilateral deprivation on the left side. This resulted in a homogenous group with no significant auditory/peripheral asymmetries between the two ears, other than the unilateral stimulation/deprivation factor of interest here. Due to advancements in CI technology over the period of inter‐implant delay, CI devices and electrode design differed between the two ears (see Table 1 for details). Cortical responses to both unilateral stimulation (left ear only and right ear only) and bilateral stimulation (both left and right ear together) were collected for all participants at initial activation of the second implant (baseline) and at a minimum of one follow‐up time point over the first year of bilateral CI use (see Figure 1). Two participants were subsequently excluded due to many noisy and/or zero‐signal flat electroencephalogram (EEG) channels (~40%) identified during the first stage of data preprocessing. Device, clinical and demographic characteristics for the 13 patients (five females) included in the study are provided in Table 1. Prospective patient recruitment and data collection took place at the Hospital for Sick Children between 2011 and 2012, and data were analyzed between 2019 and 2021.

**TABLE 1:** Participant demographics and clinical characteristics

Participant (ordered by age at CI‐1)	Age at onset of bilateral deafness (0 = birth)	Duration of bilateral deafness	Age at CI‐1/right ear	Electrode array CI‐1/right ear	Age at CI‐2/left ear	Electrode array CI‐2/left ear	Inter‐implant delay	Etiology
1	0.63	0.42	1.05	CI24R(CS)	9.67	CI513	8.62	Connexin mutation
2	0.00	1.09	1.09	CI24RE(CA)	10.33	CI24R(CS)	9.24	Unknown
3	0.00	1.73	1.73	CI24(CA)	13.36	CI513	11.63	Heredofamilial
4	0.00	1.92	1.92	CI24RE(CA)	9.82	CI24(CA)	7.91	Connexin mutation
5	0.58	1.45	2.04	CI24(CA)	10.05	CI24RE(CA)	8.02	Unknown
6	0.00	2.47	2.47	CI24M	15.99	CI513	13.52	Unknown
7	0.00	2.57	2.57	CI24(CA)	9.66	CI24RE(CA)	7.09	Unknown
8	0.00	2.69	2.69	CI24R(CS)	11.52	CI513	8.82	Heredofamilial
9	2.11	1.22	3.34	CI24R(CS)	13.25	CI24RE(CA)	9.92	Heredofamilial
10	0.00	3.38	3.38	CI24M	16.80	CI24RE(CA)	13.42	Heredofamilial
11	0.00	3.39	3.39	Not available	15.99	CI24RE(CA)	12.60	Unknown
12	0.00	3.95	3.95	CI24R(CS)	12.77	CI513	8.81	Heredofamilial
13	0.00	4.79	4.79	CI24M	16.15	CI513	11.36	Pneumococcal meningitis
Summary	Mean (SD)	Mean (SD)	Mean (SD)	(*n*)	Mean (SD)	(*n*)	Mean (SD)	(*n*)
	0.26 (0.60)	2.39 (1.25)	2.65 (1.10)	Pre‐curved (9) Straight (3) Not available (1)	12.72 (2.77)	Pre‐curved (13)	10.07 (2.19)	Heredofamilial (5)
								Unknown (5)
								Connexin mutation (2)
								Pneumococcal meningitis (1)

*Note*: All age and duration data are presented in years. Electrode arrays in order of chronology (older to newer versions of devices): CI24M—straight; CI24R(CS)—pre‐curved; CI24RE(CA)—pre‐curved; CI513—pre‐curved.

### 2.2 EEG acquisition

EEG measures were recorded longitudinally in each CI user. Figure 1 illustrates the timeline of recordings acquired for each participant following activation of their second CI (Weeks 1–2/baseline = 8.7 ± 3.3 days; Months 1–2 = 42.4 ± 6.7 days; Months 3–4 = 100.5 ± 9.5 days; Months 6–14 = 285.1 ± 85.5 days). Recordings from 62 cephalic electrodes referenced to a separate reference electrode on the right earlobe were obtained in line with previously reported protocols (Jiwani et al., 2016). Cortical responses were evoked by biphasic electrical pulses presented at 250 pulses per second in trains of 36 ms at a rate of 1 Hz via a research processor to an apical electrode (#20) of the implanted electrode array, either from each left and right CI alone (unilateral stimulation) or from both CIs together (bilaterally). Further details of stimulation and current levels are available in Data S1. Participants passively listened to the stimuli whilst watching a muted movie of choice with subtitles. Responses were recorded using the NeuroScan‐4.3 system and Synamps‐II amplifier (Compumedics USA, Inc.) with a sampling rate of 1000 Hz and an online bandpass filter of 0.15–100 Hz. Continuous EEG data were separated into 1000 ms epochs from 200 ms pre‐stimulus to 800 ms poststimulus. Epochs with activity greater than ±100 μV between 100 and 800 ms were rejected. Online analysis of the first 100 epochs/average evoked potential at electrode Cz was used to confirm that a visually identifiable waveform was present. This was repeated so that at least two visually replicable cortical responses with a minimum of 100 epochs/average were obtained for each stimulation condition. We aimed to collect a minimum of 400 epochs for each ear/stimulation condition, although in practice this was not always possible across all participants and testing sessions. The resultant responses were pre‐processed offline in MATLAB R2019b. Data were filtered from 1 to 30 Hz and recordings were re‐referenced to a common average reference. Each epoch obtained was visually inspected for ocular artifacts (eye blinks and horizontal eye movement at frontal channels) or other noise; if present, these epochs were removed for all channels. On average, 197 ± 83 epochs per stimulation condition per participant were included for further analysis. In rare cases, “bad” channels identified visually to have consistent noise over the length of the recording (such as electrodes with high impedance) were excluded from analyses.

### 2.3 Source localization

Data pre‐processing and source localization was performed in MATLAB R2019b using the Field‐Trip toolbox (Oostenveld et al., 2011) and custom scripts (available upon request). The cortical generators of the evoked responses were examined using the Time‐Restricted Artifact and Coherent source Suppression (TRACS) beamformer imaging method (Gordon et al., 2013; Wong & Gordon, 2009) (further details in Data S1). Age‐dependent head geometry and conductivities of the brain, skull and scalp were accounted for by a three‐layer boundary element model that was constructed from age‐appropriate Montreal Neurologic Institute magnetic resonance imaging templates generated with the Template‐O‐matic toolbox (Wilke et al., 2008). Activity in each voxel was normalized relative to the pre‐stimulus baseline (− 200 to −80 ms) using a pseudo‐Z statistic (PZ), calculated as a ratio of the mean signal to the standard deviation of the pre‐stimulus baseline. A one‐tailed omnibus *t* test (Petersson et al., 1999) was also conducted to determine a statistical threshold PZ of baseline brain activity (omnibus value, see Data S1). This omnibus value was subtracted from the PZ value in each voxel to identify voxels with higher than baseline brain activity. The voxel with the largest omnibus‐corrected PZ in the left (*x* ≤ −55) and right (*x* ≥ 55) auditory cortical areas (−35 ≤ *y* ≤ 5; −10 ≤ *z* ≤ 20) was extracted for subsequent statistical analyses.

### 2.4 Cortical indices

Peak omnibus‐corrected PZ values from the chosen voxels were used to calculate indices of the hemispheric representations of source activity as follows:

#### 2.4.1 Cortical lateralization

Cortical lateralization (%) characterized the hemispheric difference in activity between the left and right auditory cortices for each stimulation condition, and was defined as

(1) $$\left[\frac{\left(\text{Right auditory cortex response}-\text{Left auditory cortex response}\right)}{\left(\text{Right auditory cortex response}+\text{Left auditory cortex response}\;\right)}\right]\times 100.$$

Thus, positive values indicate greater activation in the right compared to the left auditory cortex, normalized for the overall level of activation (i.e., right‐hemispheric lateralization) and negative values indicate left‐hemispheric lateralization.

#### 2.4.2 Cortical representation of input

Cortical representation of input (%) characterized the difference in activity to input from the left and right ear for each hemisphere. Cortical representation was calculated separately for both the left and right hemisphere and defined as

(2) $$\left[\frac{\left(\text{Right}\;\mathrm{ear}\;\text{evoked response}-\text{Left}\;\mathrm{ear}\;\text{evoked response}\right)}{\left(\text{Right}\;\mathrm{ear}\;\text{evoked response}+\text{Left}\;\mathrm{ear}\;\text{evoked response}\;\right)}\right]\times 100.$$

Thus, positive values indicate greater cortical representation of input from the right (CI‐1) ear compared to the left (CI‐2) ear, normalized for the overall level of responsiveness to auditory input. Negative values therefore indicate greater cortical representation of the left ear. This metric cannot be calculated for bilateral stimulation conditions. For children in which stimulation evoked activity with PZ value below threshold in one auditory cortex, PZ was set to zero (i.e., representing that there was an absence of significant cortical response) to calculate the normalized hemispheric indices.

#### 2.4.3 Bilateral enhancement

Bilateral enhancement, a measure of bilateral processing in the left and right auditory cortex (AC), was characterized as the bilateral‐unilateral response difference in the ipsilateral hemisphere. Bilateral enhancement was calculated separately for both the left and right hemisphere and defined as the difference between the bilaterally evoked response and the unilaterally evoked response from the ipsilateral ear

(3) LeftACbilateral enhancement=Bilateral evoked response−Leftearunilateral evoked response,

(4) RightACbilateral enhancement=Bilateral evoked response−Rightearunilateral evoked response.

Thus, a positive response difference indicates that bilateral stimulation evokes greater activity in that side of auditory cortex compared to unilateral stimulation, reflecting typical bilateral response enhancement in the ipsilateral cortex during bilateral processing.

### 2.5 Speech perception

All available unilateral and bilateral speech perception scores were obtained from clinical records as a measure of functional outcome. Speech perception was assessed using the Phonemic Balanced Kindergarten monosyllabic words open‐set speech test in quiet, presented free‐field at 60 or 65 dB sound pressure level (see Data S1 for further details). Data were collected by the patient's audiologist at their clinical appointments if time permitted and were available for 11 (85%) of the participants at an average of 16.5 months after bilateral implantation. Auditory performance was scored as the percentage of words correctly identified. Bilateral benefit, the difference between bilateral and unilateral performance, was calculated to determine the amount of benefit that each ear provides when listening in bilateral conditions. As the amount of benefit that could possibly be obtained from each ear in bilateral conditions differs depending on unilateral performance in the opposite ear, a normalized measure provided an estimate of the benefit obtained as a percentage of possibly attainable benefit. Normalized bilateral benefit was calculated as follows:

(5) $$\text{Right}\;\mathrm{ear}\;\text{normalized bilateral benefit}=\left[\frac{\left(\text{Bilateral speech perception}-\text{Left}\;\mathrm{ear}\;\text{speech perception}\right)}{\left(100-\text{Left}\;\mathrm{ear}\;\text{speech perception}\;\right)}\right]\times 100,$$

(6) $$\begin{array}{l} \text{Left}\;\mathrm{ear}\;\text{normalized bilateral benefit}\\ \;=\left[\frac{\left(\text{Bilateral speech perception}-\text{Right}\;\mathrm{ear}\;\text{speech perception}\right)}{\left(100-\text{Right}\;\mathrm{ear}\;\text{speech perception}\right)}\right]\times 100. \end{array}$$

### 2.6 Statistical analyses

#### 2.6.1 Linear mixed‐effects regression models (LMMs)

Statistical analyses were performed with R v4.0.3 (R Core Team, 2020) using the lmerTest (Kuznetsova et al., 2017) and lme4 (Bates et al., 2015) packages. Data were visualized Matlab and in R using color‐blind‐friendly palettes (Garnier, 2018). LMMs were used to evaluate the variance in amplitude of cortical activation (omnibus‐corrected PZ), cortical lateralization, cortical representation, and speech perception. Fixed predictors included: time as a categorical factor (see Data S1, Weeks 1–2, Months 1–2, Months 3–4, Months 6–14), side of auditory cortex (left, right), stimulation condition (left/CI‐2, right/CI‐1, bilateral/CI‐B), and related interactions. Individual differences in cortical indices and trajectories were examined by modeling random effects of intercept and slope, respectively, and a random effect of intercept was retained in the final models (see Data S1). Covariates included age at CI‐1 (years) and duration of inter‐implant delay (years). Age at time of testing showed no significant effects on cortical indices examined here and so was not included as a covariate in the final models to avoid multicollinearity (see Data S1). Post hoc contrasts of the estimated marginal means (EMMs) were conducted using the emmeans package (Lenth, 2021). Post hoc contrasts of EMMs were completed using the Tukey method to correct for multiple comparisons (confidence level used: 0.95). Analyses of variance and pairwise post hoc analyses were implemented using the Satterthwaite and Kenward–Roger methods, respectively, to estimate denominator degrees of freedom for *t* statistics of the mixed models. Significance was defined at *P* < 0.05.

## 3 RESULTS

### 3.1 Left‐hemispheric bias for new, established, and bilateral inputs indexes cortical asymmetry

#### 3.1.1 Cortical waveforms and surface topographies

**FIGURE 2:**
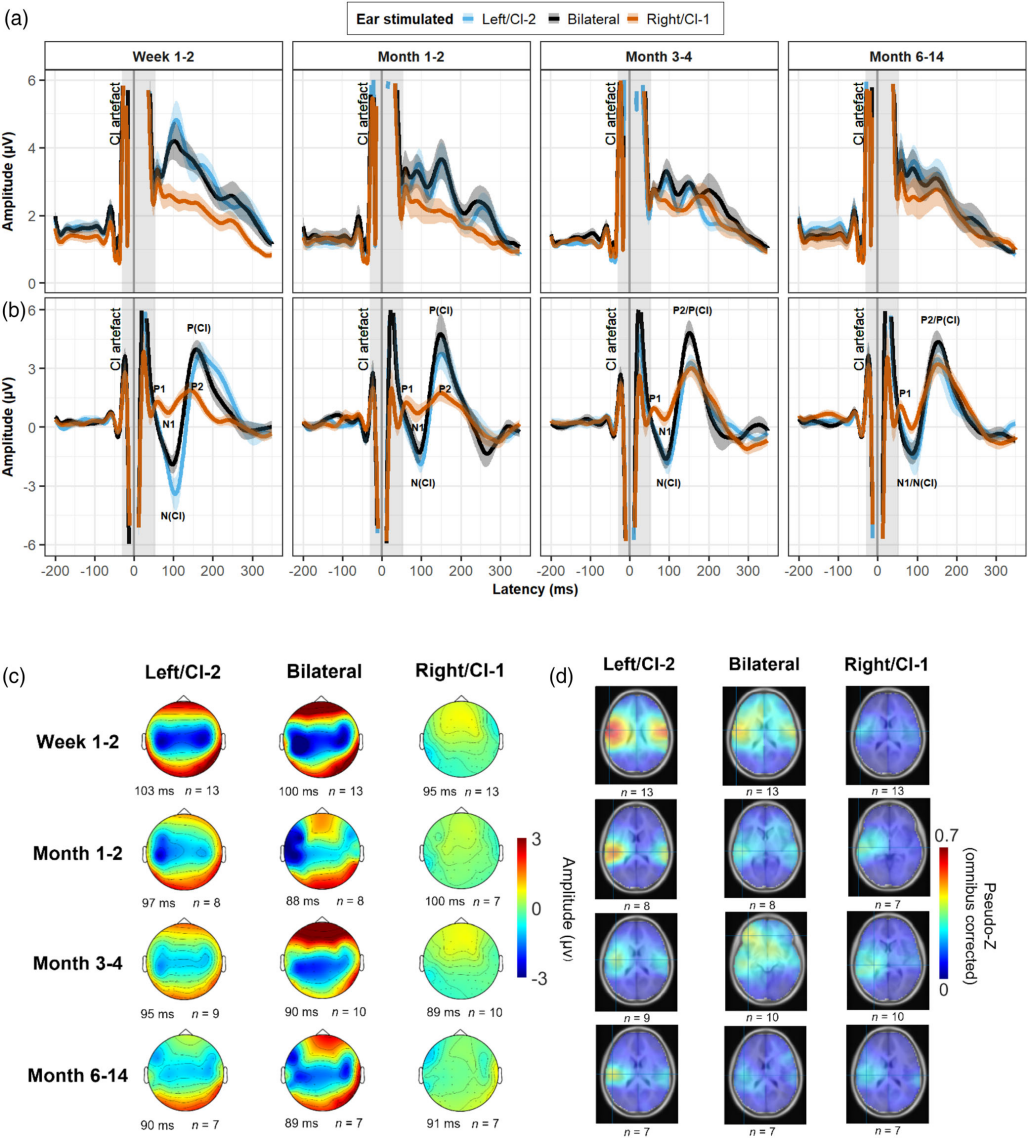
Evoked potentials with increased bilateral CI use. (a) Global field power (GFP) and (b) group mean surface potentials at the vertex recording channel (Cz) are shown for unilateral and bilateral stimulation, paneled by duration of bilateral CI use. Solid line indicates mean and shaded region ± SE. (c) Topographic distributions of mean activity across the surface of the head at the peak latency of the first identifiable component in the GFP. For each child, surface activity was averaged across a 5‐ms time window centered on the peak latency of the first component. Mean peak latency is indicated for each stimulation condition at each time point. (d) Axial view of mean evoked source activity in ~64,000 voxels evaluated using the TRACs beamformer (higher signal‐to‐noise ratio, omnibus‐corrected pseudo‐Z, in red). The highest level of activation is consistently seen in the left auditory cortex regardless of the stimulation condition or time point

Cortical responses evoked by unilateral and bilateral stimulation are shown in Figure 2 (global field power [GFP] across electrodes in Figure 2a and at Cz in Figure 2b). Morphology of the surface response measured at electrode Cz across all time points shows mature‐like P1‐N1‐P2 complex evoked by unilateral stimulation of the first‐implanted ear, typical to that of normal hearing adolescents (Jiwani et al., 2013, 2016; Yamazaki et al., 2018). In contrast, unilateral stimulation of the long‐deprived ear evoked an atypical, abnormally large “N(CI)‐P(CI)” complex in the absence of a P1 component, consistent with previous reports (Jiwani et al., 2016). Whilst the amplitudes of these peaks appeared to reduce over time with increased CI‐2 use, the atypical N(CI)‐P(CI) complex persisted and a mature P1‐N1‐P2 response remained absent. This atypical morphology was also observed in the auditory cortical response to bilateral stimulation, which more closely mirrored responses from the newly implanted ear than the first implanted ear across all time points. The first visually identifiable component in the GFP waveform (Figure 2a) following the stimulus‐related CI artifact was identified (i.e., N1/N(CI) indicated in Figure 2b) and taken forward for further analysis. Topographic surface distributions of mean evoked activity across all recording channels are shown in Figure 2c for the peak latencies of the first N1/N(CI) components (mean latency across time points, CI‐1: 93.6 ± 12.9 ms; CI‐2: 97.2 ± 11.3 ms; CI‐B: 92.7 ± 13.1 ms; see Data S1 for qualitative description of topographies).

#### 3.1.2 Source localization

**FIGURE 3:**
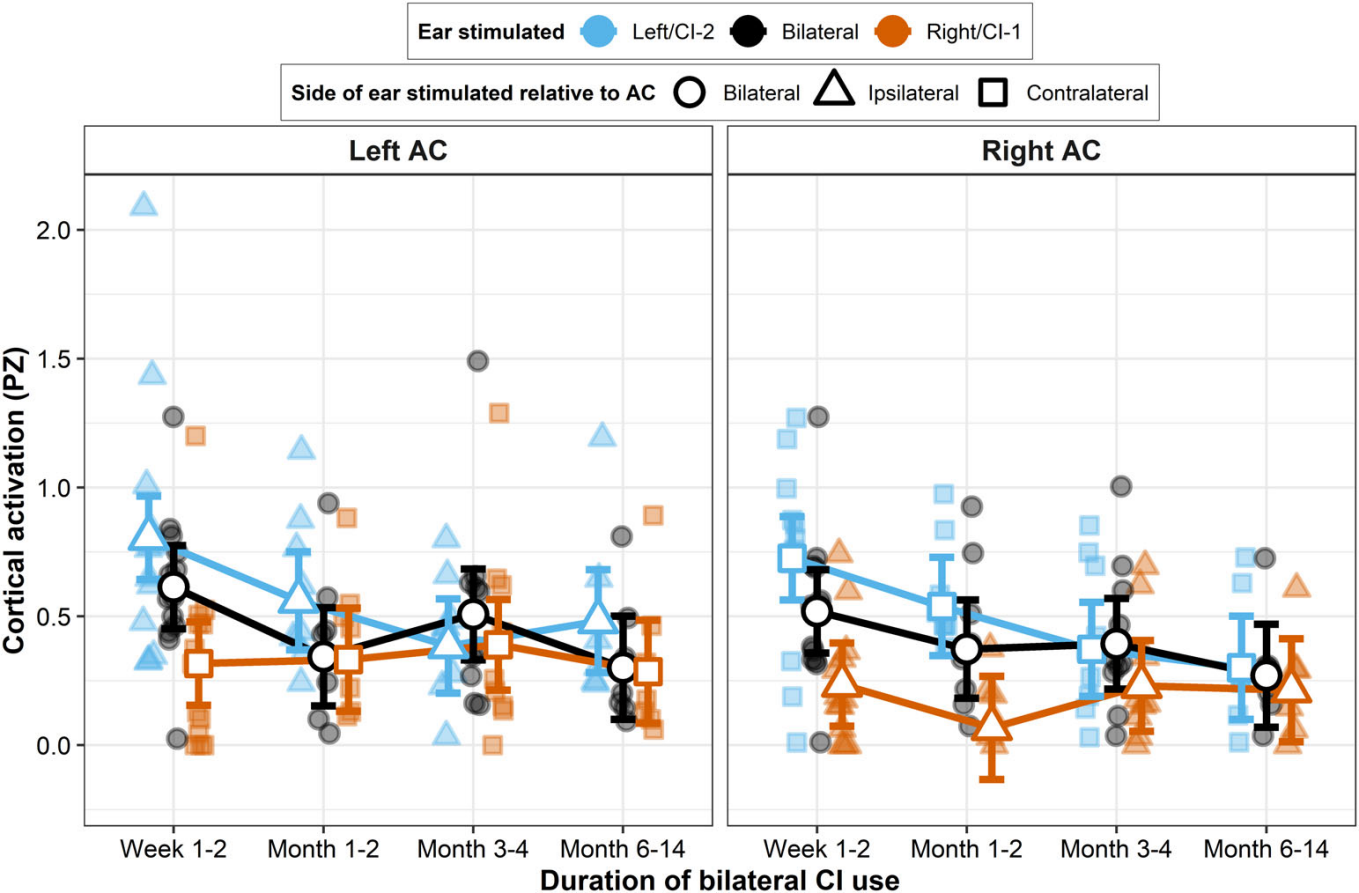
Changes in amplitude of auditory cortical activity with increased bilateral CI use. Amplitude of cortical activation (omnibus‐corrected pseudo‐Z) plotted for each child, with estimated marginal means from the linear mixed‐effect regression model (white‐filled points) and bars representing ±1 SE

Source activity underlying the N1/N(CI) surface component was examined. Group‐mean response strength (omnibus‐corrected PZ) across the brain is shown in axial view in Figure 2d. Both unilateral and bilateral auditory stimulation evoked hotspots of activity across temporal auditory regions with the most significant activation localized to the contralateral left auditory cortex. This left hemispheric bias was evident across all time points as the duration of bilateral CI use increased. One exception was seen at Months 3–4, where the most significant activation evoked by bilateral stimulation was localized to the left prefrontal cortex. An LMM examined the effects of stimulation condition, hemisphere, and time on the level of cortical activation. LMM results are summarized in Table 2 (Model 1) and the observed response strengths (PZ) are plotted along with EMMs from the model in Figure 3a. The model indicated no significant effect of age at CI‐1 or duration of inter‐implant delay on the strength of cortical activation (age at CI‐1: *F*(1,13.4) = 0.19, *p* = .67; delay: *F*(1,13.0) = 3.18, *p* = 0.10), and the random effect of intercept accounted for 36% of the model's total variance. A maximal model indicated that a random effect of slope accounted for 0.12% of the total variance and was not specified in the final model (see Data S1). An effect of hemisphere confirmed significantly greater activation in the left compared to the right AC (*F*(1, 211.2) = 9.33, *p* = .003). Variability in baseline cortical responsiveness to new input in the left auditory cortex was not predicted by age at CI‐1 (*F*(1,9) = 0.06, *p* = .81) nor the duration of inter‐implant delay (*F*(1,9) = 1.48, *p* = .25). However, level of cortical responsiveness to long‐established inputs did significantly, positively predict level of responsivity to new‐established inputs (*F*(1,9) = 14.48, *p* = s.004, see also Data S1).

**TABLE 2:** Summary of linear mixed model results for the main effects and interactions of interest on all cortical indices

Model: dependent variable	*F*	*df*	*p*	Variance (SD)
Model 1: cortical response strength (Pseudo‐Z)				
Main effects and interactions				
Time*	12.11	3, 216.09	**2.35 e−07**	
Condition*	26.32	2, 211.26	**6.23 e−11**	
Hemisphere*	9.33	1, 211.20	**.0025**	
Time × condition*	4.67	6, 211.31	**.00018**	
Time × hemisphere	0.01	3, 211.20	1.00	
Condition × hemisphere	0.88	2, 211.20	.41	
Time × condition × hemisphere	0.78	6, 211.20	.58	
Inter‐implant delay	3.18	1, 13.04	.098	
Age at CI‐1	0.19	1, 13.36	.67	
Random effects				
Intercept (participant)				0.026 (0.16)
Residual				0.046 (0.21)
Model 2: cortical lateralization (%)				
Main effects and interactions				
Time	0.65	3, 101	.58	
Condition	2.67	2, 92.87	.075	
Time x condition*	2.42	6, 92.76	**.032**	
Inter‐implant delay	0.13	1, 11.59	.73	
Age at CI‐1	0.10	1, 14.91	.75	
Random effects				
Intercept (participant)				98.08 (9.90)
Residual				1021.86 (31.97)
Model 3: cortical representation (%)				
Main effects and interactions				
Time*	7.81	3, 60.44	**.00017**	
Hemisphere	3.06	1, 58.56	.085	
Time × hemisphere*	3.48	3, 58.56	**.021**	
Inter‐implant delay	4.71	1, 12.17	.051	
Age at CI‐1	0.43	1, 13.42	.53	
Random effects				
Intercept (participant)				974.4 (31.22)
Residual				579.6 (24.07)
Model 4: bilateral enhancement (pseudo‐Z)				
Main effects and interactions				
Time	1.44	3, 74	.24	
Hemisphere*	19.08	1, 74	**.00004**	
Time × hemisphere	2.61	3, 74	.058	
Inter‐implant delay	0.025	1, 74	.88	
Age at CI‐1	0.65	1, 74	.42	
Random effects				
Intercept (participant)				0.00 (0.00)
Residual				0.09 (0.30)

*Note*: All models covaried for age at CI‐1 (years) and duration of inter‐implant delay (years) and incorporated an estimate of a random effect of intercept for each participant. Time, time point of testing following activation of CI‐2 (Weeks 1–2, Months 1–2, Months 3–4, Months 6–14); condition, ear/s stimulated (right CI‐1, left/CI2, or bilateral); hemisphere, side of auditory cortex (left or right). *Variable is a significant predictor at the .05 level.

Responses elicited by stimulation of the newly implanted ear and by bilateral stimulation were significantly greater compared to the first implanted ear (*F*(2,211.3) = 26.32, *p* = 6.227e−11; post hoc, CI‐2 > CI‐1: *t*(237) = 6.81, *p* < .0001; CI‐B > CI‐1: *t*(237) = 4.08, *p* = .0002), and responses from the newly implanted ear were significantly greater than bilateral‐evoked responses (CI‐2 > CI‐B: *t*(237) = 2.78, *p* = .016). Post hoc contrasts indicated that responses to the new input and to bilateral stimulation were significantly greater than the first implanted ear at baseline (CI‐2 > CI‐1: *t*(237) = 7.76, *p* < .0001; CI‐B > CI‐1: *t*(237) = 4.60, *p* < .0001). A significant time × condition interaction (*F*(6211.3) = 4.67, *p* = .0002) showed that these abnormally large responses decreased significantly over time, whereas responses from the first implanted ear remained stable across testing sessions. Post hoc contrasts indicated that a reduction in response amplitude occurred rapidly and was already evident by Months 1–2 of bilateral CI use (CI‐2: *t*(239) = 2.94, *p* = .019; CI‐B: *t*(239) = 2.84, *p* = .026; CI‐1: *t*(239) = 0.99, *p* = .75). No further significant changes occurred beyond Months 1–2. Consequential to reductions in response amplitude, responses elicited by stimulation of the newly implanted ear were no longer significantly larger than responses from the first implanted ear or bilateral stimulation by Months 3–4 (CI‐2 > CI‐1: *t*(237) = 0.93, *p* = .62; CI‐2 > CI‐B: *t*(237) = −0.97, *p* = .60). Responses evoked by bilateral stimulation were no longer significantly larger than responses from the first implanted ear from Months 1–2 (*t*(237) = 1.90, *p* = .14).

### 3.2 Atypical left‐hemispheric lateralization is not resolved following provision of bilateral input

**FIGURE 4:**
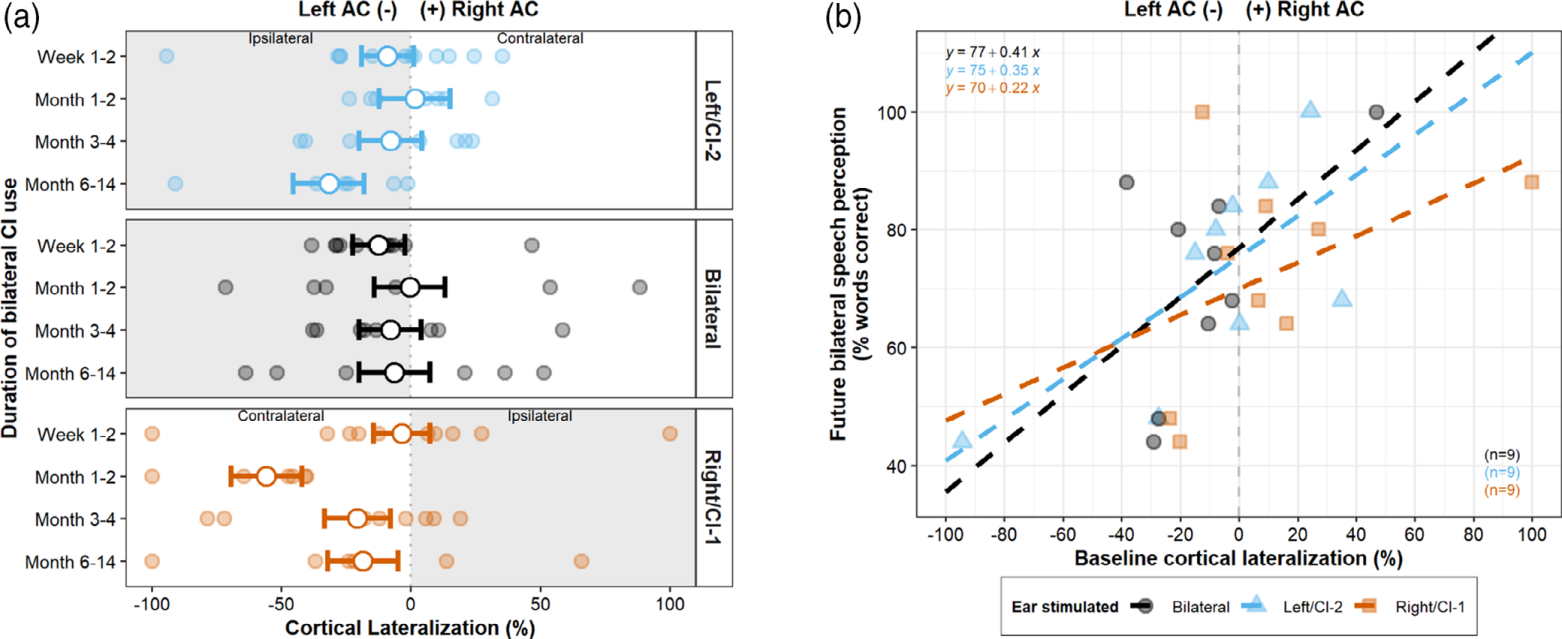
Cortical lateralization and bilateral speech perception. (a) Cortical lateralization (%) with increased bilateral CI use plotted for each child, with estimated marginal means from the linear mixed‐effect regression model (white‐filled points) and bars representing ±1 SE. (b) Stronger atypical left‐hemispheric lateralization at baseline is associated with poorer bilateral speech perception ability in the future (mean ± SD = 17.2 ± 18.6 months post‐CI‐2)

Cortical lateralization was assessed to compare relative levels of activation across the left and right AC. An LMM covarying for age at CI‐1 and inter‐implant delay provided EMMs for each condition and time point. LMM results are summarized in Table 2 (Model 2) and EMMs plotted in Figure 4a. Age at CI‐1 and duration of inter‐implant delay were not significant predictors of cortical lateralization (age at CI‐1: *F*(1,14.91) = 0.10, *p* = .75; delay: *F*(1,11.59) = 0.13, *p* = .73), and the random effect of intercept accounted for 8.8% of the model's total variance. A maximal model indicated that a random effect of slope accounted for 0.13% of the variance and was not specified in the final model. Cortical lateralization of responses evoked by either unilateral or bilateral stimulation conditions varied but, on average across time points, were more strongly lateralized to the left AC reflected by negative values (EMM ± SE cortical lateralization, CI‐1: −24.5 ± 7.1%, CI‐2: −11.7 ± 6.9%, CI‐B: −6.8 ± 6.9%). There was a nonsignificant effect of condition (*F*(2,92.9) = 2.67, *p* = .075) and time (*F*(3101) = 0.65, *p* = .58) on cortical lateralization. However, a significant time × condition interaction did indicate that activity was significantly more left‐lateralized for CI‐1 at the Months 1–2 time point (*F*(6,92.8) = 2.42, *p* = .032; post hoc contrasts, CI‐B > CI‐1: *t*(105) = 3.04, *p* = .008; CI‐2 > CI‐1: *t*(104) = 3.15, *p* = .006), and that CI‐1 responses became more strongly left‐lateralized between baseline and Months 1–2 of bilateral CI use (post hoc contrast: *t*(110) = −3.12, *p* = .012).

We next explored whether an overall atypical imbalance across stimulation conditions toward the left hemisphere influenced bilateral processing. A linear model covarying for age at CI‐1 and inter‐implant delay indicated that overall baseline cortical lateralization (collapsed across conditions) was a significant predictor of future bilateral speech perception (*F*(1,19) = 11.9, *p* = .003) indicating greater left‐lateralized cortical responses were associated with poorer bilateral speech perception. Whilst this relationship did not significantly differ between stimulation conditions (*F*(2,19) = 0.15, *p* = .86), Figure 4b illustrates that a stronger relationship between left‐lateralized responses to bilateral stimulation and bilateral speech perception may have most strongly contributed to this overall effect. Age at CI‐1 was also a significant predictor of bilateral speech perception (*F*(1,19) = 5.4, *p* = .031), indicating that those implanted early in childhood had better bilateral speech perception. There was no significant effect of duration of inter‐implant delay (*F*(1,19) = 3, *p* = .10) or stimulation condition (*F*(2,19) = 0.56, *p* = .58).

### 3.3 Cortical overrepresentation of new input increases with a longer duration of unilateral deprivation

**FIGURE 5:**
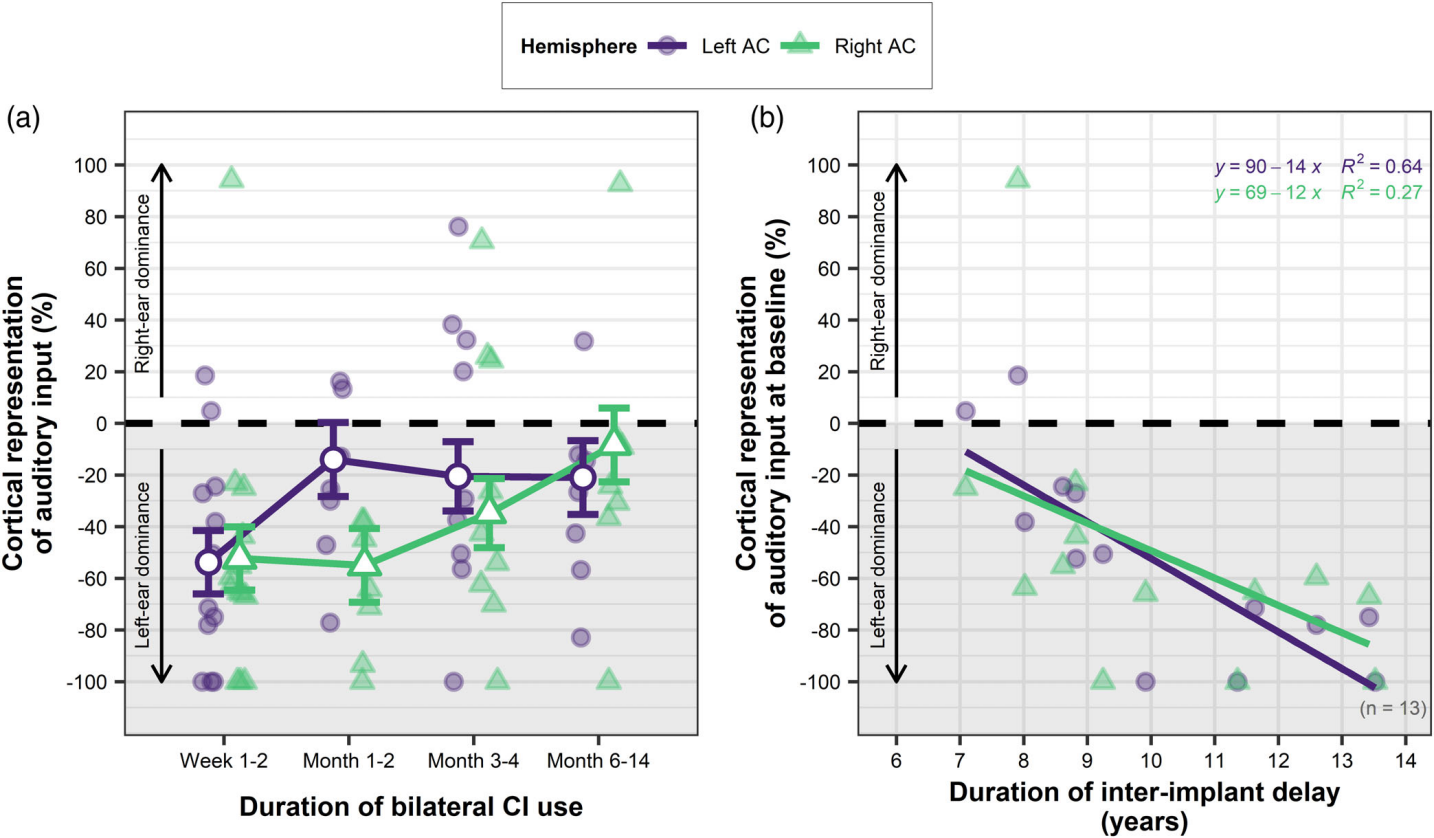
Asymmetrical cortical representation of auditory inputs and duration of unilateral deprivation. (a) Cortical representation (%) of new and established inputs in the auditory cortices, illustrating dominance of the left newly implanted ear in both auditory cortices. Values are plotted for each child as a function of increased bilateral CI use with estimated marginal means from the linear mixed‐effect regression model (white‐filled points) and bars representing ±1 SE. (b) Greater asymmetrical cortical representation of new inputs from the left ear at initial CI‐2 use is predicted by a longer duration of unilateral deprivation/stimulation

Cortical representation of input was assessed to compare how strongly each ear was represented in the auditory cortex relative to the other ear. A LMM covarying for age at CI‐1 and inter‐implant delay provided EMMs for each hemisphere and time point. LMM results are summarized in Table 2 (Model 3) and EMMs plotted in Figure 5a. Age at CI‐1 was not a significant predictor of cortical representation across hemispheres and time points (*F*(1,13.4) = 0.43, *p* = .53) and a random effect of intercept accounted for 62.7% of the model's total variance. A maximal model indicated that a random effect of slope accounted for 0.25% of the variance and was not specified in the final model. Contrary to our hypothesis, most adolescents showed significantly greater cortical representation of the newly implanted ear (“left ear dominance” shown in Figure 5a) as opposed to the first implanted ear in both auditory cortices (median, left AC = −33.6%, right AC = −49.4%; one‐sample *t* tests on EMMs, left AC: *t*(21.1) = −2.48, *p* = .044; right AC: *t*(21.1) = −3.41, *p* = .005, Bonferroni corrected). Cortical representation of input was negatively predicted by the duration of inter‐implant delay and this effect was at the threshold for significance when collapsed across hemispheres and time points (*F*(1,12.2) = 4.71, *p* = .051). A significant effect of time (*F*(3,60.4) = 7.81, *p* = .0002) and time × hemisphere interaction (*F*(3,58.6) = 3.48, *p* = .021) indicated that this abnormally strong representation of the newly implanted ear decreased significantly over time from baseline to Months 6–14 in both the left and right AC (post hoc contrasts, left AC: *t*(68) = −2.63, *p* = .050; right AC: *t*(68) = −3.54, *p* = .004), and from baseline to Months 3–4 in the left AC only (*t*(67.8) = −2.92, *p* = .024). Despite the significant decrease in cortical representation of new inputs relative to established inputs over time, Figure 5a illustrates that an abnormal representation of the newly implanted ear in both auditory cortices did not resolve for the majority of adolescents following six or more months of bilateral CI use (median cortical representation: left AC = −26.6%, right AC = −24.3%).

A multiple linear regression model was used to examine possible predictors of the highly variable level of initial cortical representation. Greater cortical representation of new input at baseline was not explained by the atypically high absolute response levels to CI‐2 stimulation (left AC: *F*(1,9) = 0.53, *p* = .49; right AC: *F*(1,9) = 1.57, *p* = .24), indicating that those adolescents with higher absolute response levels to new input did not necessarily show stronger cortical representation of the newly implanted ear relative to the first implanted ear. Age at CI‐1 was not a significant predictor of cortical representation (left AC: *F*(1,9) = 0.77, *p* = .40; right AC: *F*(1,9) = 0.07, *p* = .80). Cortical representation of auditory input in both hemispheres was significantly predicted by the duration of inter‐implant delay (Figure 5b, left AC: *F*(1,9) = 16.75, *p* = .003; right AC: *F*(1,9) = 6.78, *p* = .029; controlling for age at CI‐1 and baseline response amplitude). Figure 5b illustrates that a greater cortical representation of inputs from the newly implanted ear (“left ear dominance”) was strongly associated with a longer duration of inter‐implant delay, particularly in the ipsilateral left auditory cortex.

### 3.4 Bilateral processing is disrupted in the left auditory cortex

**FIGURE 6:**
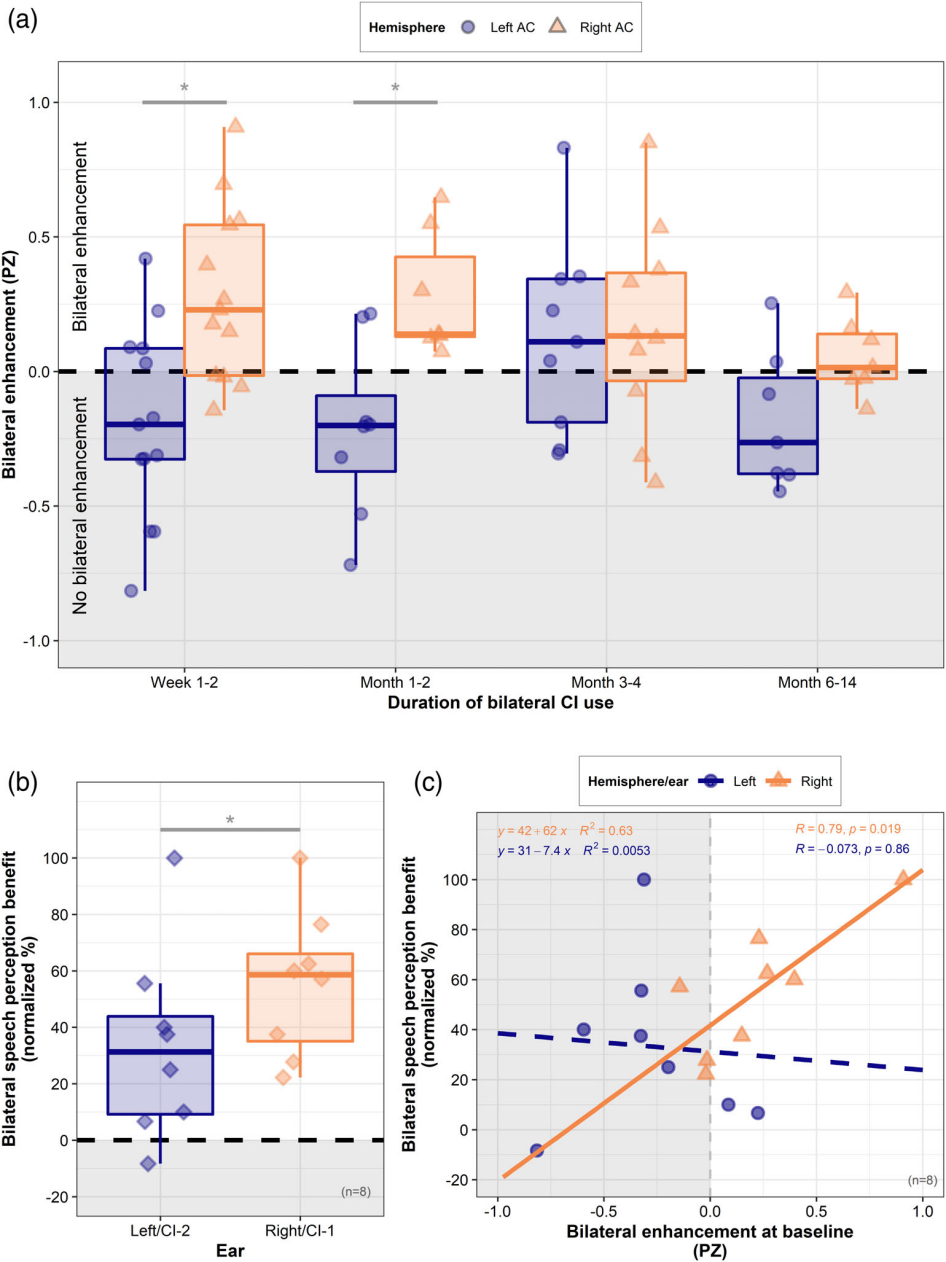
Bilateral response enhancement and bilateral speech perception benefit. (a) Bilateral enhancement (omnibus‐corrected pseudo‐Z) in the left and right auditory cortex over time. Grey lines indicate significant post hoc Tukey HSD comparisons (*p < .005, **p < .001). (b) Amount of improvement in speech perception provided by each ear in bilateral conditions (improvement in speech perception that each ear provided in bilateral listening conditions, compared to when listening with the opposite ear only) as a proportion of possible improvement. Grey lines indicate significant paired *t* test comparisons (*p < .05). (c) Associations between bilateral response enhancement in the left and right auditory cortex and bilateral speech perception benefit obtained by the ipsilateral ear

Bilateral enhancement for each hemisphere and time point is plotted in Figure 6a and illustrates that bilateral enhancement was evident in the ipsilateral right AC in most adolescents. Conversely, this typical bilateral enhancement effect was absent in the left AC for most adolescents. A LMM controlling for duration of inter‐implant delay and age at CI‐1 (results summarized in Table 2, Model 4) demonstrated an effect of hemisphere, confirming significantly reduced bilateral enhancement in the left AC compared to the right AC across time points (*F*(1,74) = 19.1, *p* = 4.021e−05). There was no significant effect of time across hemispheres (*F*(3,74) = 1.44, *p* = .24), indicating that bilateral enhancement remained stable in the right AC (post hoc contrast, Weeks 1–2 – Months 6–14: *t*(78.5) = 0.03, *p* = 1), and did not develop over time in the left AC (post hoc contrast, Weeks 1–2 to Months 6–14: *t*(78.5) = 1.59, *p* = .39). A hemisphere × time interaction approached significance (*F*(3,74) = 2.61, *p* = .058) with post hoc investigations indicating that the level of bilateral enhancement no longer significantly differed between the left and right AC after 3 months of bilateral CI use (Weeks 1–2: *t*(73.2) = 3.77, *p* = .0003; Months 1–2: *t*(74.6) = 3.04, *p* = .003; Months 3–4: *t*(74.3) = 0.22, *p* = .83; Months 6–14: *t*(73.2) = 1.38, *p* = .17), although bilateral enhancement was still absent in the left AC in most adolescents at the latest Months 6–14 follow‐up time point (median (IQR) = −0.26 (0.36)). There was no significant effect of duration of inter‐implant delay (*F*(1,74) = 0.025, *p* = .88) or age at CI‐1 (*F*(1,74) = 0.65, *p* = .42), and a random effect of intercept did not account for any of the model's variance (see Data S1).

Figure 6b shows the amount of bilateral speech perception benefit provided by each ear, normalized as a percentage of total possible benefit (see Data S1 for un‐normalized bilateral benefit data). The level of bilateral benefit varied substantially, but, on average, the amount of benefit adolescents obtained from their second CI in bilateral listening conditions was significantly lower than that from their first CI (paired *t* test, mean difference = 22.2%, *t*(7) = 3.43, *p* = .011). Reduced bilateral benefit from left/CI‐2 was not explained by poorer unilateral speech perception for this ear (*F*(1,6) = 0.06, *p* = .82). A linear model covarying for age at CI‐1 and inter‐implant delay indicated that the amount of bilateral speech perception benefit was significantly predicted by level of cortical bilateral enhancement at baseline (collapsed across left and right sides: *F*(1,10) = 6.02, *p* = .033), indicating greater bilateral enhancement in the ipsilateral auditory cortex was associated with greater bilateral speech perception benefit for this ear. Whilst this relationship did not significantly differ between the left and right side (perhaps due to the limited sample size, *F*(1,10) = 2.6, *p* = .14), linear trends explored in Figure 6c illustrate that a positive association between bilateral enhancement in the right auditory cortex and bilateral benefit from the right ear drove this overall effect. Age at CI‐1 was a significant predictor of bilateral speech perception benefit across both ears (*F*(1,10) = 9.19, *p* = .012), indicating that those implanted early in childhood obtained more bilateral speech perception benefit from their devices. There was no significant effect of duration of inter‐implant delay (*F*(1,10) = 2.47, *p* = .15).

## 4 DISCUSSION

Here we determine the impact of long‐term unilateral deprivation/stimulation in early childhood on cortical bilateral processing following the restoration of bilateral input into adolescence. The study identified cortical imbalances and disruptions to bilateral processing that may compromise effective integration of inputs from both ears. Five main findings are relevant to understanding the cortical mechanisms underlying restoration of bilateral auditory function during pre‐/adolescence following early and profound long‐term, left‐sided unilateral deprivation: (1) activation was more significant in the ipsilateral left than contralateral right auditory cortex, creating a left‐hemispheric asymmetry across all unilateral and bilateral conditions that predicted poorer bilateral speech perception abilities; (2) new auditory inputs from the second‐implanted ear were more highly represented in the auditory cortex than established inputs from the first‐implanted ear, indicating a cortical imbalance and greater weighting toward the long‐deprived ear that increased with a longer duration of unilateral deprivation/stimulation during early development; (3) bilateral processing mechanisms were altered in the left auditory cortex and suggested a decreased ability to inhibit and balance the dominant ipsilateral input from the newly implanted ear with that of the first‐implanted ear when listening bilaterally; (4) dynamic and rapid changes to new auditory input were observed early in the auditory pathways even beyond an early sensitive period for bilateral input; (5) despite rapid initial changes in amplitude of cortical response to new inputs, left‐hemispheric asymmetries, bilateral processing mechanisms, and cortical overrepresentation of the newly implanted ear did not fully resolve with increased duration of bilateral experience to reach those of a typical balanced cortical state. This study demonstrates the potential for neuroplastic adaptation in a highly reorganized auditory system that has developed under unilateral conditions and occurring beyond an early sensitive period. The rapid changes in cortical responses observed here could be interpreted as an attempt of the adolescent auditory system to reach a balanced state, although this was not fully realized over the period that we observed. These findings provide evidence at the cortical level of an imbalance between inputs from the first‐ and second‐implanted ear predicted by increased delay in the restoration of bilateral hearing in deaf adolescents. These cortical imbalances toward greater weighting of input from the second CI may manifest as common patient‐reported experiences including loud and conflicting input from the second CI compared to the first CI alone (Emond et al., 2013; Low et al., 2020). Such outcomes may be limited by restricting the length of inter‐implant delay (Gordon et al., 2013; Han & Dimitrijevic, 2015; Polonenko et al., 2018a). In cases where long delays cannot be avoided, pre‐operative counseling should include the likelihood for: (i) a protracted period of adaptation to the second CI; (ii) cortical overrepresentation of the newly implanted ear; and (iii) the potentially irreversible imbalance between new and established inputs that, combined, may limit benefits of bilateral CI use. Postoperatively, clinicians should aim to balance input delivered by the two CIs and provide rehabilitative techniques to support listening with both implants together.

### 4.1 Left‐hemispheric asymmetry driven by long‐term unilateral CI use compromises bilateral processing

Our findings demonstrate typical contralateral dominance in the left auditory cortex for stimulation of the right first implanted ear, consistent with the well‐established contralateral bias of the auditory system (Langers et al., 2005; Pantev et al., 1986; Suzuki et al., 2002) and with our previously published data in both children and adolescents with normal hearing (Easwar et al., 2017; Gordon et al., 2013; Polonenko et al., 2018a; Yamazaki et al., 2018) and right‐ear unilateral CI users (Gordon et al., 2013; Jiwani et al., 2016). When contralateral activity is stronger than ipsilateral activity for both ears, auditory pathways are essentially symmetric. In contrast to typical cortical symmetries, an ipsilateral dominance was found for stimulation of the left, second‐implanted ear in the present cohort, with the most significant activation located in the left auditory cortex, and a leftward lateralization of cortical responses reflecting stronger ipsilateral compared to contralateral activity. Stronger ipsilateral activation of the left auditory cortex by the newly implanted ear was significantly associated with stronger contralateral activation of the left auditory cortex by the first implanted ear but was not explained by the duration of unilateral deprivation/stimulation or the age at CI‐1. Therefore, ipsilateral dominance to newly restored auditory input appears to reflect greater responsivity of the left auditory cortex due to contralateral strengthening from long‐term right ear stimulation rather than cortical immaturity associated with the duration of deafness experienced. In consequence, for bilateral stimulation, the typical symmetrical pattern of activity with a rightward bias for these nonspeech sounds (Easwar et al., 2017) was absent: although patterns of activation were more bilaterally distributed compared to unilateral stimulation, bilateral‐evoked activation was most significant in the left auditory cortex and showed leftward lateralization. Greater left‐hemispheric asymmetry across both unilateral and bilateral conditions at initial bilateral activation was predictive of poorer bilateral speech perception abilities many years later. Together these findings indicate an atypical left‐hemispheric bias across both ears and a consequential asymmetric weighting of bilateral pathways that appears to be driven by long‐term unilateral CI use with long‐term consequences for bilateral speech perception.

Whilst participants were largely homogenous in their auditory and peripheral characteristics and showed no evidence of audiological asymmetries between the two ears, electrode array technology did differ between the two ears. Therefore, how this device‐related asymmetry may contribute to the observed cortical asymmetry should also be considered. Due to technological advancements in CI design over the long period of inter‐implant delay, we might expect that the device‐related asymmetries would benefit the left/CI‐2 ear in terms of better speech perception and promoting typical contralateral activation of right auditory cortex to achieve cortical balance. However, we saw no evidence of an increased perceptual or cortical benefit for the second compared to first implanted ear, suggesting that device‐related differences likely did not heavily contribute to these central asymmetries we observed. Although determining the influence of device‐ and peripheral‐related differences between the two ears on central processing is beyond the scope of this study, mismatches in frequency, level, and timing between the devices likely create disruptions in binaural processing (Gordon et al., 2016; Gordon, Chaikof, et al., 2012; Gordon, Salloum, et al., 2012; Goupell et al., 2013; Hu & Dietz, 2015; Kan et al., 2015). Thus, ongoing efforts to quantify and resolve device related mismatches are important.

### 4.2 Unilateral deprivation underlies cortical imbalance and an overrepresentation of new auditory input

Animal models and data from deaf children with unilateral hearing indicate that the hearing/stimulated ear becomes more extensively represented in the central auditory system, whereas central representation of the deaf ear is weakened, leading to a cortical preference for the hearing ear and absence of typical contralateral‐ear bias (Gordon et al., 2013, 2015; Kral, Hubka, et al., 2013; Popescu & Polley, 2010; Tillein et al., 2016). We therefore hypothesized that the first implanted ear would be more strongly represented compared to the second implanted ear in both auditory cortices. In direct contrast, we observed a stronger representation of the newly implanted ear compared to the first implanted ear in both hemispheres, resulting in a cortical imbalance toward the long‐deprived left ear (Figure 5). It may be possible that a device‐related advantage from the newer technology in the left ear could contribute to stronger responses and representation of input from the newly implanted ear. However, greater cortical representation of the newly implanted ear was not explained by the atypically large absolute response amplitudes from this ear, but rather was significantly predicted by a greater duration of inter‐implant delay. This suggests that unilaterally driven development compromises the ability of central auditory system to equally represent inputs from each ear. This, subsequently, likely interferes with integration of sound inputs from both ears during bilateral listening. Indeed, whilst the right auditory cortex displayed typical bilateral enhancement effects previously evidenced in both hemispheres of normal hearing controls (Easwar et al., 2017), the left auditory cortex showed significantly decreased bilateral enhancement, suggesting an inability to suppress the ipsilateral responses from the newly implanted ear. Studies have indicated that typical bilateral processing involves suppression of the unilateral response from the ipsilateral ear so that contralaterality prevails (Easwar et al., 2017; Fujiki et al., 2002) (see also for review of mammalian physiological mechanisms, Grothe et al., 2010). It is plausible that the significantly reduced bilateral enhancement observed here in the left auditory cortex reflects the degradation of typical inhibitory balance mechanisms in the long‐deprived ipsilateral pathways that, at baseline, had not yet developed functional relevance to the level of perceptual benefit obtained from the left ear in bilateral listening conditions. This compromised ability to suppress ipsilateral inputs from the newly implanted ear may explain why adolescents were unable to fully realize the functional benefits of listening with their second‐implanted left ear in bilateral conditions, even several years after bilateral input had been restored. Conversely, long‐term right‐ear stimulation likely enabled ipsilateral response inhibition to develop typically in the right hemisphere and for contralateral left‐ear bias to manifest. Indeed, we saw that greater bilateral enhancement (i.e., ipsilateral response inhibition) in the right hemisphere was associated with a higher level of perceptual benefit derived from the right ear in bilateral listening conditions. These findings provide mechanistic insights into why, despite being able to gain speech perception benefits from a second sequential implant (Galvin et al., 2007, 2010), some of these adolescents report “excessive loudness” and “conflicting input from CI‐2” compared to CI‐1 (Low et al., 2020), reduced long‐term daily device usage compared to CI‐1 (Low et al., 2020), and deficits in binaural fusion (Steel et al., 2015) and sound localization (Reeder et al., 2017) that rely on the efficient integration of bilateral inputs.

The current findings pertain to the early stages of cortical auditory processing (here, the N1/N1(CI) component) in response to nonspeech stimuli under passive listening conditions, and thus demonstrate a cortical imbalance in fundamental auditory processing of low‐level acoustic features, such as frequency and intensity, that are known to be reflected in the early typical N1 component in hearing individuals (Dimitrijevic et al., 2008, 2009). Whilst studying this most basic level of auditory processing, upon which more complex processes are built, can give us an indication of how vulnerable the underlying pathways may be to later processing and perceptual difficulties, it would be important to also examine higher order responses to complex speech stimuli and known neural markers of active speech processing such as the P2 component (Han et al., 2020). Individuals with CIs may be able to work around these lower level vulnerabilities that we identify here by developing strategies such as increasing attention and listening effort, using semantic and situational context, and exploiting information from visual cues to aid successful spoken communication. As such, although we believe the early N1/N1(CI) component examined here reflects primary auditory processing, it is possible that stronger activation of the auditory cortex by stimulation of the newly implanted ear could be modulated by and reflect contributions of inputs from disparate nonauditory cortical regions. For instance, research has demonstrated the facilitative role of the visual modality (Anderson et al., 2017; Isaiah et al., 2014) and recruitment of the visual cortex (Giraud et al., 2001) in adult CI rehabilitation, the involvement of nonsensory left frontal regions in deaf children with CIs (Easwar et al., 2017; Lee et al., 2007) that likely reflects increased auditory attention and listening effort (Lawrence et al., 2018; Wijayasiri et al., 2017; Wild et al., 2012), and increased functional connectivity between occipital and frontal cortical regions (Smieja et al., 2020). Here, approximately 3 months after bilateral implantation, we observed the most significant activation to bilateral stimulation localized to the left frontal lobe (Figure 2d). This may reflect the increased task difficulty experienced with binaural fusion in children with CIs (Steel et al., 2015) and the recruitment of higher level nonsensory areas to facilitate resolution of new and established inputs together following unilaterally driven development. Future work examining possible functional connectivity between auditory and extratemporal regions will be important to gain a comprehensive understanding of the neural networks involved in successful adaptation to new auditory input and neuroplastic changes in the auditory pathways following long‐term unilateral deprivation.

### 4.3 Cortical asymmetries do not fully resolve over the first year despite rapid initial changes

Longitudinal investigation within the same participants enabled us to determine the effects of introducing bilateral inputs to the auditory system that developed with only unilateral stimulation, over the first year of bilateral implantation. Whilst responses evoked by the newly implanted ear were atypically stronger than those of the first implanted ear and bilateral stimulation, response amplitude reduced rapidly over the initial 1–2 months followed by a plateau over the remainder of year. Responses elicited by the first implanted ear remained stable over time and a significant interaction confirmed that reductions in response amplitudes were specific to the newly implanted ear and bilateral stimulation. This suggests the observed changes reflect cortical adaptation to new auditory input rather than being attributable to other factors that vary across testing sessions over time, such as age‐related changes and measurement error. Evidence of initial rapid reductions in evoked responses indicate that dynamic change is possible in the adolescent auditory system, consistent with the widely demonstrated notion of adolescence as a period of substantial cortical development. Furthermore, evidence suggests that sensitive periods might be extended if a system has not gathered all necessary or reliable information (for review see Frankenhuis & Walasek, 2020). Such a possibility may explain the seemingly high potential for plastic adaptation here. However, despite the decline in response amplitudes to newly established auditory input, atypical response morphologies, cortical asymmetries, cortical representation of input, and bilateral processing mechanisms remained unresolved, suggesting that limitations on the adaptive capacity of the unilaterally developed auditory system existed in this group of adolescents. The cortical trajectories observed here mirror longitudinal behavioral trajectories in sequentially implanted adolescents (Reeder et al., 2017) where initial significant improvements in speech perception in quiet for CI‐2 are seen over the first 3 months of bilateral CI use, followed by only subtle improvements that leave poorer performance in the CI‐2 ear than the CI‐1 ear up to 24 months later (Jiwani et al., 2016; Reeder et al., 2017).

A limitation of our study is the lack of longer term follow to enable modeling of changes beyond the first year of bilateral implantation: it is possible that, depending upon the level of plasticity, the auditory system could have the potential to achieve a balanced state and successful integration of new auditory input beyond the first year of rehabilitation later in adolescence. However, given existing cohort data demonstrating unilaterally driven reorganization of the auditory system is present following 3–4 years of bilateral cochlear implant experience (Gordon et al., 2013) and that bilateral listening difficulties continue for these children 2–7 years after bilateral implantation (Illg et al., 2019; Polonenko et al., 2018b; Reeder et al., 2017), we would anticipate that any further changes would be limited. Nevertheless, longer term follow‐up would be necessary to determine if unilaterally driven changes remain unresolved later in adolescence and even in adulthood. It is also possible that the effect of reaching typical cortical processing over time was not detected here at the group level due to the limited follow‐up sample size of available patients and/or known heterogeneity of the adolescent brain and trajectories of neurodevelopmental change (see review in Foulkes & Blakemore, 2018). Here, we also evaluated individual differences in trajectories (i.e., slopes) for all cortical indices and identified remarkably similar trajectories within this group of adolescents despite large and significant individual differences in baseline cortical levels (i.e., intercepts). Similarities between individuals' trajectories observed here likely reflect the homogeneity of this group (i.e., similar ages at CI‐1 and CI‐2 and all with a long duration of inter‐implant delay) and suggest uniform and a somewhat restricted level of adaptation to bilateral auditory input once an early sensitive period for bilateral integration has passed.

It is also important to note that our study sample comprised adolescents all with left‐sided deafness and a left‐ear sequential implant. Whilst outside the scope of this study, an investigation of adolescents with right‐sided deafness and a right‐ear sequential implant would be necessary to understand whether the side of deafness and sequential implantation has a differential impact on central auditory plasticity and to increase the generalizability of our current results. Whilst our sample represent a unique sequentially implanted cohort, one similarity between these adolescents with congenital/early onset bilateral deafness who receive a single CI early in life, and children with congenital/early‐onset unilateral “single‐sided deafness” (SSD) is that the auditory system develops under unilateral, asymmetric hearing conditions in both these cohorts before bilateral stimulation is later provided. Previous research with children with early onset SSD shows that a strong cortical preference for the hearing, acoustically stimulated ear develops (Lee et al., 2020; Polonenko et al., 2017). When bilateral input is provided with limited delay via a CI in the deaf ear, representation of electrical input from this ear is promoted and a balanced cortical representation of electrical and acoustic inputs from each ear can be established (Lee et al., 2020; Polonenko et al., 2017). This compares to the current findings where, following the provision of bilateral input via a second CI, a balanced representation of established electrical (CI‐1) and novel electrical inputs (CI‐2) from each ear is not established in the auditory cortices. Taken together, these studies could suggest that the auditory system may face unique challenges to bilateral than unilateral deafness. However, the cortical findings above were from studies of children with short durations of single sided deafness (<3 years), which differs to the long duration of unilateral hearing experienced by the current cohort (approximately 10 years). Future studies would need to directly compare these and other cohorts to determine the differential impact of the hearing loss, and the duration of unilateral stimulation prior to bilateral stimulation on the potential for cortical adaptation in the auditory system.

## CONFLICT OF INTERESTS

Karen A. Gordon: SickKids Foundation funds, speaker's bureau Cochlear Corp., Investigator, Cochlear Corp.‐sponsored clinical trials at SickKids, Lecturer at Salus University. The other authors declare no conflict of interests.

## ETHICS STATEMENT

Patient and/or parental written informed consent was obtained according to the Declaration of Helsinki under study protocol #1000002954 approved by the Hospital for Sick Children Research Ethics Board.

## Supporting information


**Data S1:** Supporting Information

## Data Availability

Pseudonymized EEG and speech perception data that support the findings of this study are publicly available in Open Science Framework at at http://doi.org/10.17605/OSF.IO/Q4BCN, https://osf.io/q4bcn/. All identifying demographic and clinical information have been removed to protect the participants' privacy and are available upon reasonable request from the corresponding author.
