# Supplementary material for: Cortical imbalance following delayed restoration of bilateral hearing in deaf adolescents
Source: Hum Brain Mapp. 2022 Apr 15;43(12):3662–79. doi: 10.1002/hbm.25875 (PMC9294307; doi:10.1002/hbm.25875)
Supplement: Supplementary file 1 — Data S1: Supporting Information [file HBM-43-3662-s001.docx]

# Supporting information

## Supplementary methods

### 1. Participants

Cortical responses to unilateral-only stimulation at initial activation of the second implant (i.e., baseline time point) have been previously reported for the current sample who formed part of a larger cohort in Jiwani et al., (2016). Here, for the first time, we report cortical responses to bilateral stimulation at baseline and longitudinal follow-up recordings within the same individuals.

All children with access to the Canadian publicly funded health care system can be considered for cochlear implantation in our clinical centre at the Hospital for Sick Children. All cochlear implant candidacy decisions are based on multi-disciplinary assessment and all children receiving CIs at our centre are invited to participate in EEG research measures, thus helping to reduces bias of socio-economic factors and sex. Furthermore, there is no evidence of sex bias in prevalence of childhood hearing loss or cochlear implantation. All participants received Cochlear Ltd. devices.

### 2. Stimuli

Unilateral stimulation levels were at the upper part of the dynamic range (i.e., maximum comfortably loud levels) and balanced between sides as determined by assessing amplitudes of the electrically auditory evoked brainstem response (EABR). This procedure has been reported previously (Easwar et al., 2017; Gordon et al., 2013; Jiwani et al., 2016). In brief, the maximum amplitude of the EABR wave eV that remained comfortable for the participant to listen to on each side was determined, and current intensities that resulted in similar EABR wave eV amplitudes on each side were the basis of intensities used for cortical recordings. As stimulation was provided via a research processor to a single apical electrode (#20) for both EABR and cortical recordings, rather than using the participant’s processor, their MAP threshold and comfortable ranges were not considered. On average, current levels used for stimulation were 213.3 ± 13.6 µA for CI-1, 200.9 ± 9.3 µA for CI-2, and the unilateral levels were used together in the bilateral condition. Current levels used did not significantly differ across testing sessions (CI-1: *F*(3,25.2) = 1.55, *P* = 0.23; CI-2: *F*(3,25.1) = 1.44, *P* = 0.25), nor did they predict cortical response amplitudes elicited by stimulation of the respective ear (CI-1, Left AC: *F*(1,28.8) = 1.18, *P* = 0.29; CI-1, Right AC: *F*(1,25.8) = 0.005, *P* = 0.95; CI-2, Left AC: *F*(1,18.4) = 1.30, *P* = 0.27; CI-2, Right AC: *F*(1,16.7) = 0.09, *P* = 0.76).

### 3. Source localization

The Time-Restricted Artifact and Coherent source Suppression (TRACS) beamformer imaging method is an adaptive spatial filter and a variant of the linearly constrained minimum variance beamformer. It accounts for the electrical artefact generated by the CI during auditory stimulation and supresses up to 97% of the artefact (Wong & Gordon, 2009). Here, a window of -30 to 55ms was used to define the CI artefact based on visual inspection of artefact latency in the surface waveforms. Following artefact suppression, TRACS estimates the contribution (dipole activity) of each of the 3 × 3 × 3 mm voxels (approximately 64,000) of the brain’s volume to the recorded surface activity. Source activity in each hemisphere was then evaluated while suppressing activity in the contralateral hemisphere, as both auditory cortices could have coherent activity. Full details of the TRACS method are available in Gordon et al., (2013) and Wong & Gordon (2009).

A one-tailed omnibus t-test (Petersson et al., 1999) was conducted in which half of the trials were flipped in polarity to produce a plus-minus average (which removes the time-locked activity) to determine a statistical threshold PZ of baseline brain activity (omnibus value). Peak dipole strength and the corresponding latency were estimated from the source time series for all voxels. The ten voxels with the largest PZ above omnibus threshold were identified in both the left (x ≤  -55) and right (x ≥ 55) auditory cortical areas ( -35 ≤ y ≤ 5; -10 ≤ z ≤ 20) (Gordon et al., 2013; Jiwani et al., 2016; Lee et al., 2020; Polonenko et al., 2017) before the voxel with the largest omnibus-corrected PZ in the left and right auditory cortex was extracted for statistical analyses.

### 4. Speech perception

Speech perception was assessed free-field in a sound-treated audiometric booth. A PBK list of 25 words were presented through a centrally located loudspeaker (0-degrees azimuth) using monitored-live voice at 65dB sound pressure level through a GSI-61 Grason-Stadler audiometer (Jiwani et al., 2016). Participants listened under unilateral conditions while wearing only their right CI (*n*=11, median(IQR) = 10(10)months), their left CI (*n*=10, median(IQR) = 9(9)months) and under bilateral conditions while wearing both devices at the same time (*n*=9, median(IQR) = 8(7)months). When multiple speech perception assessments were available, scores from the first available test date were used, giving the smallest spread and most consistency in time since activation of the second implant (median(IQR) = 9(10)months; range = 6 – 55months).

### 5. Statistical analyses

#### 5.1 Time as a categorical vs continuous factor

The longitudinal design of the study was categorical in nature, with participants asked to attend EEG sessions in the first week after initial activation of their CI-2, and then following one month, three months, six months and twelve months of CI-2 use. Time was therefore treated as a categorical factor (illustrated in Fig. 1) and missing data was treated as missing at random, with reasons including lack of time after clinical appointments, inability to take time away from school, and moving away from the area. All available EEG data were included in the analyses. Separate models specifying time as a continuous rather than categorical factor confirmed no differences in the effects reported throughout.

#### 5.2 Developmental effects and covarying for age

Whilst it is common to control for the effects of age in developmental neuroimaging studies, as these adolescents were profoundly deaf before receiving their first CI, the duration of inter-implant delay is more representative of their time-in-sound and auditory cortical development compared to their chronological age. Furthermore, age at time of testing and duration of inter-implant delay were strongly and significantly correlated (*R* = .93, *P*= 5.24e-06) due to the similarity in age at which children received their first and second CIs. Linear mixed models specifying age rather than inter-implant delay as a covariate confirmed there were no significant effects of age (nor trends approaching significance) throughout. Therefore, to avoid multicollinearity in the final models, duration of inter-implant delay was included as a covariate and chronological age was not.

#### 5.3 Random effects estimates

Subject-related random effects (intercept and slope) were examined by comparing maximal models to simple models. Specifying subject-related random effects provides an estimate of how much an individual subject deviates from the main effect estimate and the amount of variance explained by these individual differences. We first specified a simple random-intercept model, allowing for participants to vary around the grand mean level of activation and around the fixed effects estimates. Secondly, we specified a simple random-slope model, allowing for participants to vary in their direction and rate of change in activation over time. We then specified a maximal model with a random intercept and random slope as correlated terms (i.e. not independent of each other). Time was treated as a continuous factor and all models covaried for age at CI-1 and duration of inter-implant delay. The random effects estimate from the maximal models were used to determine the proportion of total variance accounted for by each of the random effects. The simple models were also compared to the maximal model using an ANOVA. The change in loglikelihood and in AIC and BIC statistics between the models were used to determine whether specifying each random effect improved the goodness of fit and reduced the prediction error of the model. Random effects of slope explained <1% of the variability in cortical activation (results are reported throughout the main text) and so they were not included in the final models to increase parsimony. One exception was the LMM analysis of cortical bilateral enhancement where a random effect of slope explained 9% of the variability. However, a comparison of the maximal to simple models indicated that the random effect of slope did not significantly improve the model and so was not included in the final model. In this case, a random effect of intercept explained 0% of the variability but was still included in the final model to account for the non-independence of repeated measurements from each participant.

## Supplementary results

### 1. Surface topographies

For stimulation of the first implanted ear, surface topographies (Fig. 2C) indicated a consistent pattern over time of frontal-positive/posterior-negative polarity typical to that seen in normal hearing children and adolescents (Easwar et al., 2017; Gordon et al., 2013; Jiwani et al., 2016; Polonenko et al., 2018; Yamazaki et al., 2018). Stimulation of the newly implanted ear evoked an atypical central-negative polarity that was distributed bilaterally across the scalp, consistent with previous reports at the time of initial CI-2 activation (Jiwani et al., 2016). There was a tendency for the negative polarity to orientate ipsilateral to the newly implanted ear (i.e. leftward) and this pattern did not resolve with increased CI-2 experience. A similar topographical pattern was seen in response to bilateral stimulation, whereby an atypical central-negative polarity was distributed bilaterally across all time points, with stronger negativity distributed leftward and ipsilateral to the newly implanted ear.

### 2. Individual differences in initial cortical responsiveness to new auditory input

The maximal model confirmed large inter-individual differences in cortical activation levels at baseline, but negligible individual differences in change trajectories. Further to this, comparison of the maximal model with simple models indicated that specifying a random intercept significantly improved the goodness of fit of the model (Δ*χ^2^*(2) = 43.23, *P*< 4.106e-10), whereas specifying a random slope did not (Δ*χ^2^* (2) = 1.89, *P* = 0.39). Given the atypical ipsilateral left hemispheric bias, possible predictors of the initial highly variable level of cortical response to new input in the left AC were examined including age at CI-1, duration of inter-implant delay and existing cortical responsivity to established inputs (i.e. unilaterally-driven effects). A best subset linear regression analysis confirmed that baseline responsiveness to CI-1 was the ‘best’ predictor of initial responsiveness to CI-2 stimulation, explaining the most variance with the lowest prediction error compared to age and duration of inter-implant delay (*adjR^2^* = .50, *Cp* = 2.56, *BIC* = -4.91).

### 3. Baseline level of cortical response predicts overall change in activation

For responses to new CI-2 and bilateral stimulation, the ultimate level of change in amplitude from baseline to the latest available time point was not predicted by age at CI-1 (*F*(1,140) = 1.28, *P* = 0.26) nor duration of inter-implant delay (*F*(1,140) = .27, *P* = .60), but was predicted by baseline response levels (*F*(1,140) = 12.51, *P* = .0005). A negative relationship indicated a greater decrease in individuals with higher levels of response at baseline (unstandardized beta coefficient = -.40, *P* = 0.002), reflecting that a higher baseline level affords more scope for decline. A significant main effect of condition indicated that there was a greater decrease in amplitude of response elicited by stimulation of the newly implanted ear compared to bilateral stimulation (*F*(1,140) = 4.22, *P* = 0.042; post-hoc: *t*(140) = -1.97, *P* = 0.050). Regression lines from the model’s estimates incorporating effects of age at CI-1 and inter-implant delay are plotted in Supplementary Figure 1. This visualization of the model’s predictions suggests that the negative relationship observed between baseline activity and change in activity may have been largely driven by the newly implanted ear in the left AC, although a significant interaction was not observed (baseline response*condition*hemisphere: *F*(1,140) = 0.79, *P* = 0.37).

Ultimate level of change in cortical representation over time (latest available recording – baseline recording) was not predicted by age at CI-1 (*F*(1,18) = 0.33, *P* = 0.57), duration of inter-implant delay (*F*(1,18) = 0.11, *P* = 0.75), nor baseline levels of cortical representation (F(1,18) = 0.99, p = 0.33).


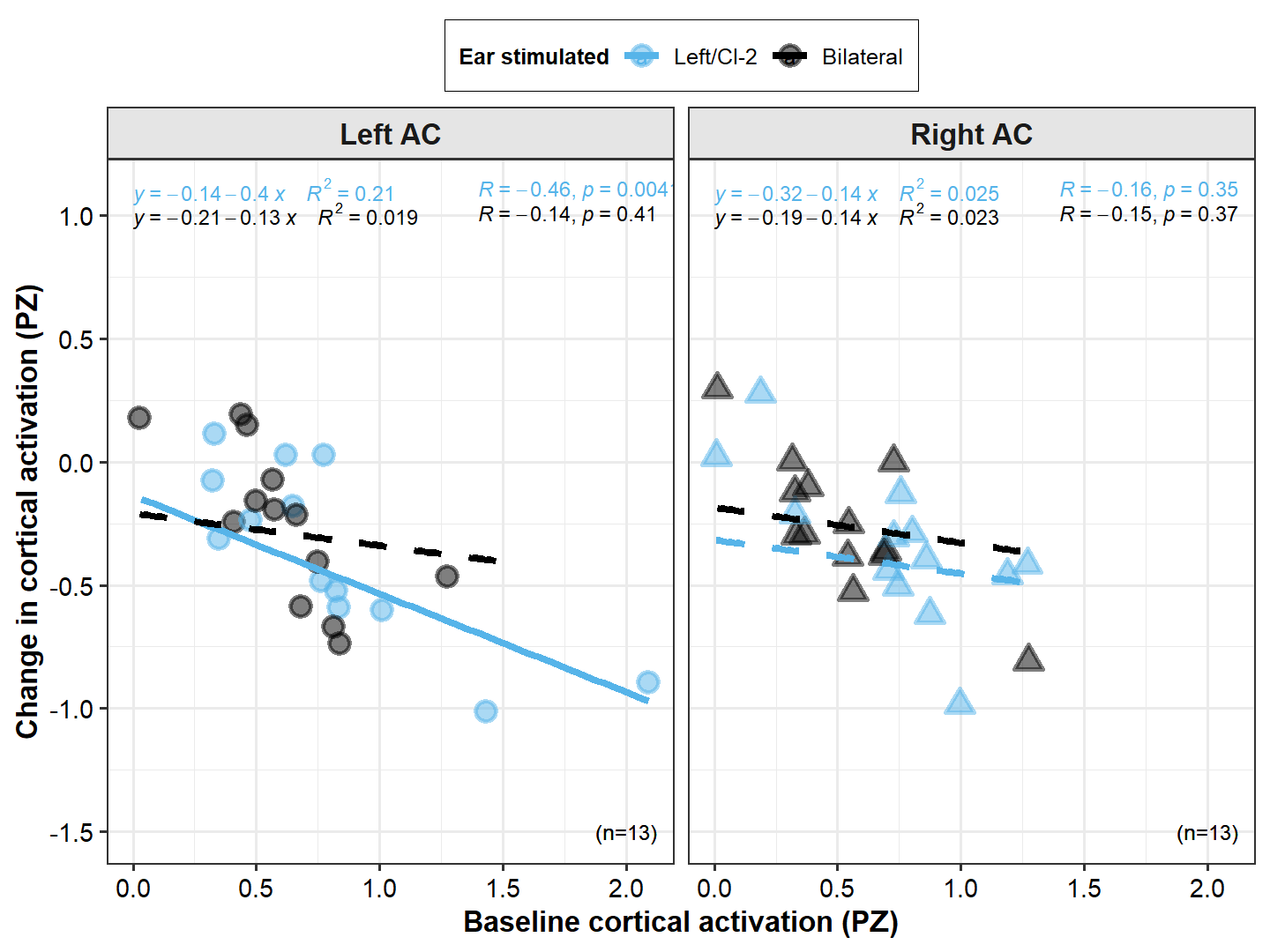


**Supplementary Figure 1:** Degree of change in cortical activation following bilateral implantation is predicted by baseline activation levels at initial CI use. Actual observations are plotted (points) with regression lines incorporating the model’s estimates of the effects of covariates.

### 4. Better functional outcomes for the first implanted ear

A paired-samples t-test of speech perception scores confirmed that the first implanted ear was the better hearing ear (mean difference = 30.4%, *t*(9) = -3.55, *P* = 0.006). Un-normalized bilateral benefit data are displayed in Supplementary Figure 2. On average, adolescents obtained significantly more benefit from their first implanted ear compared to their newly-implanted ear in bilateral listening conditions (paired t-test, mean difference = 31%, *t*(7) = 3.05, *P* = 0.019). As illustrated in Fig. 6B and Supplementary Figure 2, all adolescents obtained bilateral benefit from their first implanted right ear in bilateral listening conditions. All but one adolescent obtained some benefit from the newly implanted left ear in bilateral conditions: this adolescent had poorer speech perception in bilateral listening conditions compared to listening with their first implant alone, indicating that providing a second implant impeded their existing speech perception ability.


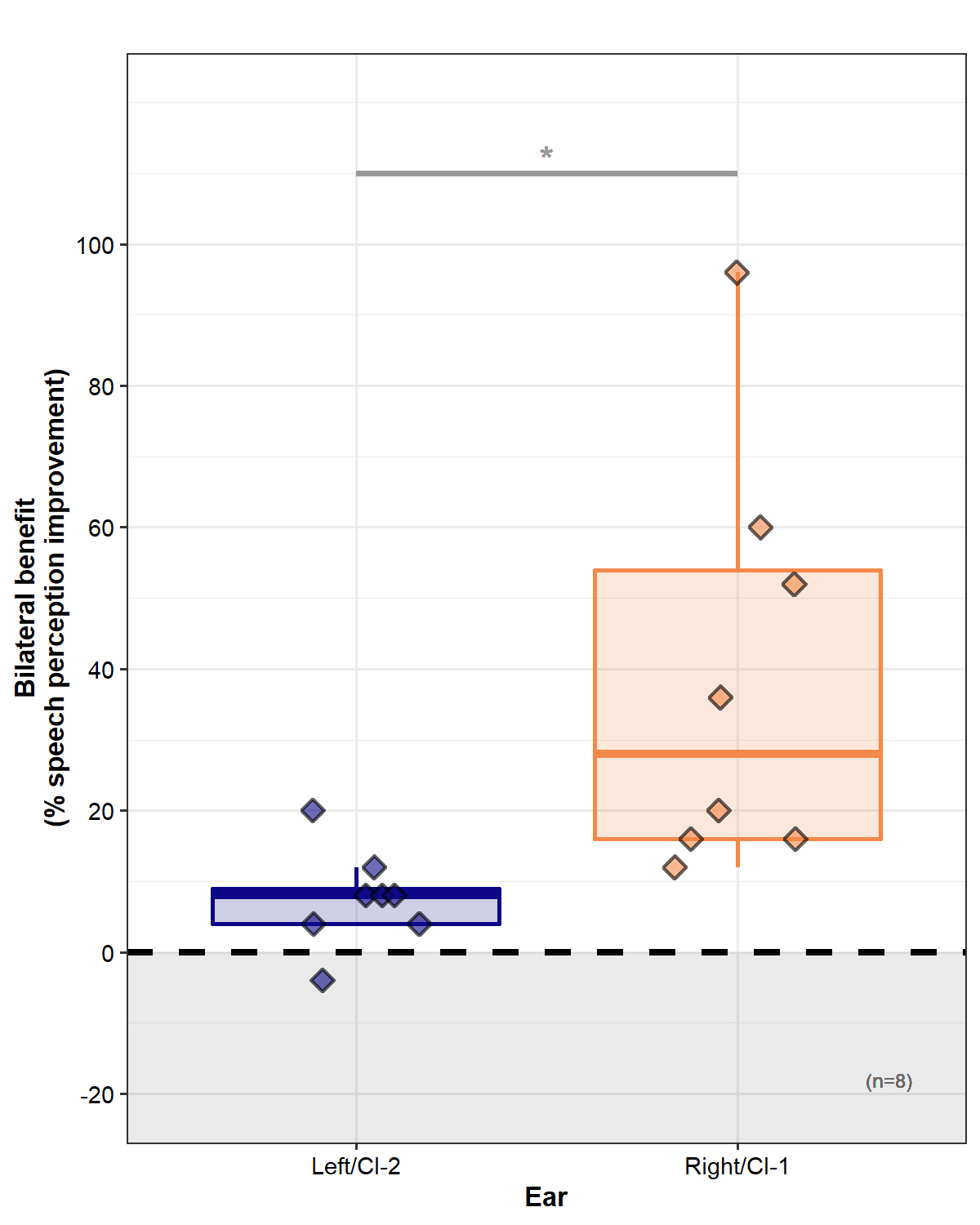


**Supplementary Figure 2:** Amount of improvement in speech perception (%) provided by each ear in bilateral conditions. **P* < .01.

## References

Easwar V, Yamazaki H, Deighton M, Papsin B, Gordon K. Simultaneous bilateral cochlear implants: Developmental advances do not yet achieve normal cortical processing. Brain Behav 2017; 7

Gordon KA, Wong DDE, Papsin BC. Bilateral input protects the cortex from unilaterally-driven reorganization in children who are deaf. Brain 2013; 136: 1609–25.

Jiwani S, Papsin BC, Gordon KA. Early unilateral cochlear implantation promotes mature cortical asymmetries in adolescents who are deaf. Hum Brain Mapp 2016; 37: 135–52.

Lee H-J, Smieja D, Polonenko MJ, Cushing SL, Papsin BC, Gordon KA. Consistent and chronic cochlear implant use partially reverses cortical effects of single sided deafness in children. Sci Rep 2020; 10: 21526.

Petersson KM, Nichols TE, Poline JB, Holmes AP. Statistical limitations in functional neuroimaging I. Non-inferential methods and statistical models. Philos Trans R Soc B Biol Sci 1999; 354: 1239–60.

Polonenko MJ, Gordon KA, Cushing SL, Papsin BC. Cortical organization restored by cochlear implantation in young children with single sided deafness. Sci Reports 2017 71 2017; 7: 1–8.

Polonenko MJ, Papsin BC, Gordon KA. Delayed access to bilateral input alters cortical organization in children with asymmetric hearing. NeuroImage Clin 2018; 17: 415–25.

Wong DDE, Gordon KA. Beamformer suppression of cochlear implant artifacts in an electroencephalography dataset. IEEE Trans Biomed Eng 2009; 56: 2851–7.

Yamazaki H, Easwar V, Polonenko MJ, Jiwani S, Wong DDE, Papsin BC, et al. Cortical hemispheric asymmetries are present at young ages and further develop into adolescence. Hum Brain Mapp 2018; 39: 941.
